# Polyunsaturated fatty acid intake and incidence of type 2 diabetes in adults: a dose response meta-analysis of cohort studies

**DOI:** 10.1186/s13098-022-00804-1

**Published:** 2022-03-03

**Authors:** Mingyuan Hu, Zhengmei Fang, Tao Zhang, Yan Chen

**Affiliations:** School of Public Health, Wannan Medical College, No. 22, Wenchang West Road, Higher Education Park, Wuhu, 241000 People’s Republic of China

**Keywords:** Polyunsaturated fatty acid, Type 2 diabetes, Dose–response, Meta-analysis

## Abstract

**Background:**

To evaluate the association and dose–response relationship between polyunsaturated fatty acid (PUFA) intake and incidence of type 2 diabetes (T2D) in adults.

**Methods:**

PubMed, Embase, Cochrane Library, and Web of Science databases were searched for cohort studies that examined the association between PUFA and T2D incidence published up to September 6, 2021. Relative risk (RR) or hazard ratio (HR) was used as the effect indicator, each effect size was expressed by 95% confidence interval (CI). The presence of heterogeneity of effect size between studies was assessed by the Q-test and I^2^ statistics. If I^2^ ≥ 50%, the random-effects model was applied, otherwise the fixed effects model was used. Sensitivity analysis was performed for all models. Potential publication bias was assessed. We conducted linear and nonlinear dose–response meta-analyses, calculated summary relative risk (SRR).

**Results:**

Twenty-five articles were selected including 54,000 patients in this study. Our estimates observed no linear associations between total PUFA and the incidence of T2D. However, the summary dose–response curve of T2D risk increased in a nonlinear pattern with the consumption of omega-3 PUFA (*P*_nonlinearity_ < 0.001) and docosahexaenoic acid (DHA) (*P*_nonlinearity_ = 0.040). Our subgroup analysis showed that total PUFA intake was associated with increased incidence of T2D in Europe (RR: 1.040, 95% CI 1.009 to 1.072), and Australia (RR: 1.188, 95% CI 1.113 to 1.269). However, total PUFA intake was associated with decreased T2D incidence in Asia (RR: 0.897, 95% CI 0.860 to 0.936). Subgroup analysis based on PUFA types showed that DHA intake was associated with decreased T2D incidence (RR: 1.164, 95% CI 1.048 to 1.294) while linoleic acid (LA) decreased T2D incidence (RR: 0.956, 95% CI 0.930 to 0.983). Regarding the sex subgroup, women’s intake of total PUFA would increase the risk of T2D (RR: 1.049, 95% CI 1.019 to 1.079) while total PUFA intake decreased the risk of T2D in men (RR: 0.955, 95% CI 0.913 to 0.999).

**Conclusion:**

For specific PUFA, dose–response curves show nonlinear significant associations between PUFA intakes and T2D. It may be necessary to pay attention to the effects of PUFA and type of intake on T2D.

*Trial registration* Not applicable

**Supplementary Information:**

The online version contains supplementary material available at 10.1186/s13098-022-00804-1.

## Background

Type 2 diabetes (T2D) accounts for 90–95% of all diabetes cases and is a complex metabolic disorder characterized by insufficient insulin secretion and hyperglycemia caused by insulin resistance (IR) [1, 2]. It is estimated that the global prevalence of T2D will increase from 171 million people in 2000 to 366 million people in 2030 [3], which will have a devastating effect on overall health [4]. T2D increases the risk for diabetes-related complications, including cardiovascular disease, nephropathy, retinopathy, microangiopathy [5], and premature death [6], and thus contributes to high healthcare costs [7]. Thereby, it may be necessary to understand the factors associated with T2D incidence for preventing and reducing adverse outcomes of T2D.

Epidemiological and clinical trial evidence demonstrates that diet plays a major role in preventing or developing T2D [4, 8, 9]. Currently, a diet low in total and animal fats and high in plant fats was recommended to prevent T2D [7]. A previous study showed that a diet rich in unsaturated fatty acid (UFA), such as the Mediterranean dietary pattern, may prevent the development of T2D [10]. Polyunsaturated fatty acid (PUFA) is a classification of UFA that contains two or more double bonds [11], which has been recommended to prevent T2D by the American Diabetes Association [12]. Evidence from a review demonstrates that PUFA has a protective effect on T2D development [13]. Omega-3 PUFA has been shown to decrease the production of inflammatory mediators, decreasing the development of T2D [14]. Omega-6 PUFA, but not omega-3 PUFA was reported to improve insulin sensitivity in a meta-analysis [15]. Specific PUFA may differ in their health effects, the association between the type of PUFA intake and the incidence of T2D merit further evaluation. Besides, from China Health and Nutrition Survey, low and moderate marine omega-3 PUFA consumption was associated with higher T2D risk whereas high marine omega-3 PUFA consumption was not associated with T2D risk [16]. Whether there is a relationship between PUFA intake at different doses and the incidence of T2D and what kind of relationship also needs to be clarified.

Herein, the objective of this study was to evaluate the association and the dose–response relationship of T2D and PUFA intake. In addition, a subgroup analysis concerning gender, geographic locations, duration of follow-up, and PUFA classifications was performed in this study to further explore the association between PUFA intake and the incidence of T2D in adults.

## Methods

### Search strategy

Published data for this meta-analysis were identified by search and selection in PubMed, Embase, Cochrane Library, and Web of Science databases from inception to September 6, 2021. Search strategy keywords included “Acids, Unsaturated Fatty” OR “Unsaturated Fatty Acids” OR “Unsaturated Fatty Acid” OR “Acid, Unsaturated Fatty” OR “Fatty Acid, Unsaturated” OR “Polyunsaturated Fatty Acids” OR “Acids, Polyunsaturated Fatty” OR “Fatty Acids, Polyunsaturated” OR “Polyunsaturated Fatty Acid” OR “Acid, Polyunsaturated Fatty” OR “Fatty Acid, Polyunsaturated” OR “Fatty Acid” OR “Fatty Acids, Esterified” OR “Esterified Fatty Acids” OR “Esterified Fatty Acid” OR “Acid, Esterified Fatty” AND “T2DM” OR “type 2 diabetes mellitus” OR “type 2 diabetes” OR “T2D”. The detailed search strategy from PubMed is listed in Additional file 1.

### Eligibility criteria

Studies were included if they met the following criteria: (1) individuals who consume PUFA; (2) individuals ≥ 18 years old; (3) cohort studies that reported the association between intake of PUFA and the incidence of T2D (The T2D diagnosis was self-reported diabetes or fasting glucose); (4) studies reported a hazard ratio (HR) or relative risk (RR) with a 95% confidence interval (CI); (5) studied published in English; (6) latest research results of the same author.

Exclusion criteria were as follows: (1) animal experiments; (2) randomized controlled trials (RCTs); (3) reviews and meta-analyses, conference articles, and letters.

### Data extraction

Data extraction was independently performed by Mingyuan Hu and Zhengmei Fang. If a discrepancy existed, a third party (Tao Zhang) would participate in the extraction of data. The extracted information included the last name of the first author, year of publication, the country where the study was conducted, duration of follow-up, number of participants, sex, age, the total number of participants, T2D assessment, exposure, PUFA intake per category, adjusted risk estimates expressed as HR, or RR with 95% CIs and adjustment factors.

### Risk of bias assessment

The Risk of bias in non-randomized studies of interventions (ROBINS-I) assessment tool [17] was used to evaluate the methodological quality of the included studies. The scale includes seven aspects: bias due to confounding, bias due to selection of participants, bias due to exposure assessment, bias due to misclassification during follow-up, bias due to missing data, bias due to measurement of results, and bias due to selective reporting of results. The overall risk of bias of each paper was categorized into “Low”, “Moderate”, and “Serious”.

### Statistical analysis

RR or HR was used as the effect indicator, each effect size was expressed by 95% CIs. The presence of heterogeneity of effect size between studies was assessed by the Q-test and I^2^ statistics. If I^2^ ≥ 50%, the random-effects model was applied, otherwise the fixed effects model was used. Models were stratified by sex, geographic location (United States, Europe, Australia, Asia), duration of follow-up (< 10 years and ≥ 10 years), and exposures of PUFA types [PUFA, omega-3, omega-6, alpha-linolenic acid (ALA), eicosapentaenoic acid (EPA), docosahexaenoic acid (DHA), EPA in combination with DHA, linoleic acid (LA), arachidonic acid (AA)] to perform the subgroup analysis. Sensitivity analysis was performed to test whether each single study could influence the stability of the results. Potential publication bias was assessed by Begg’s test. When publication bias occurred, the “cut-and-fill method” was adopted to adjust publication bias.

A linear and nonlinear trend of the dose–response relation was estimated. Each study’s specific slope (linear trend) and its standard error were calculated from the RR /HR of PUFA intake and relevant natural logarithms. Then meta-regression and restricted maximum likelihood (REML) of random effects were used to estimate the synthetically study-specific slope. The nonlinear dose–response relationship between PUFA intake and the incidence of T2D was evaluated using binary random-effects meta-regression and REML estimation. The method of restricted cubic splines with three knots at percentiles 10%, 50%, and 90% of the distribution was adopted using the generalized least-square model to synthesize the research results of two specific trends. We tested for potential nonlinearity using quadratic splines.

All analyses were performed using Stata 15.1 software (Stata Corporation, College Station, TX, USA). And *P* < 0.05 was considered to be statistically significant.

## Results

### Literature search and study characteristics

**Fig. 1:**
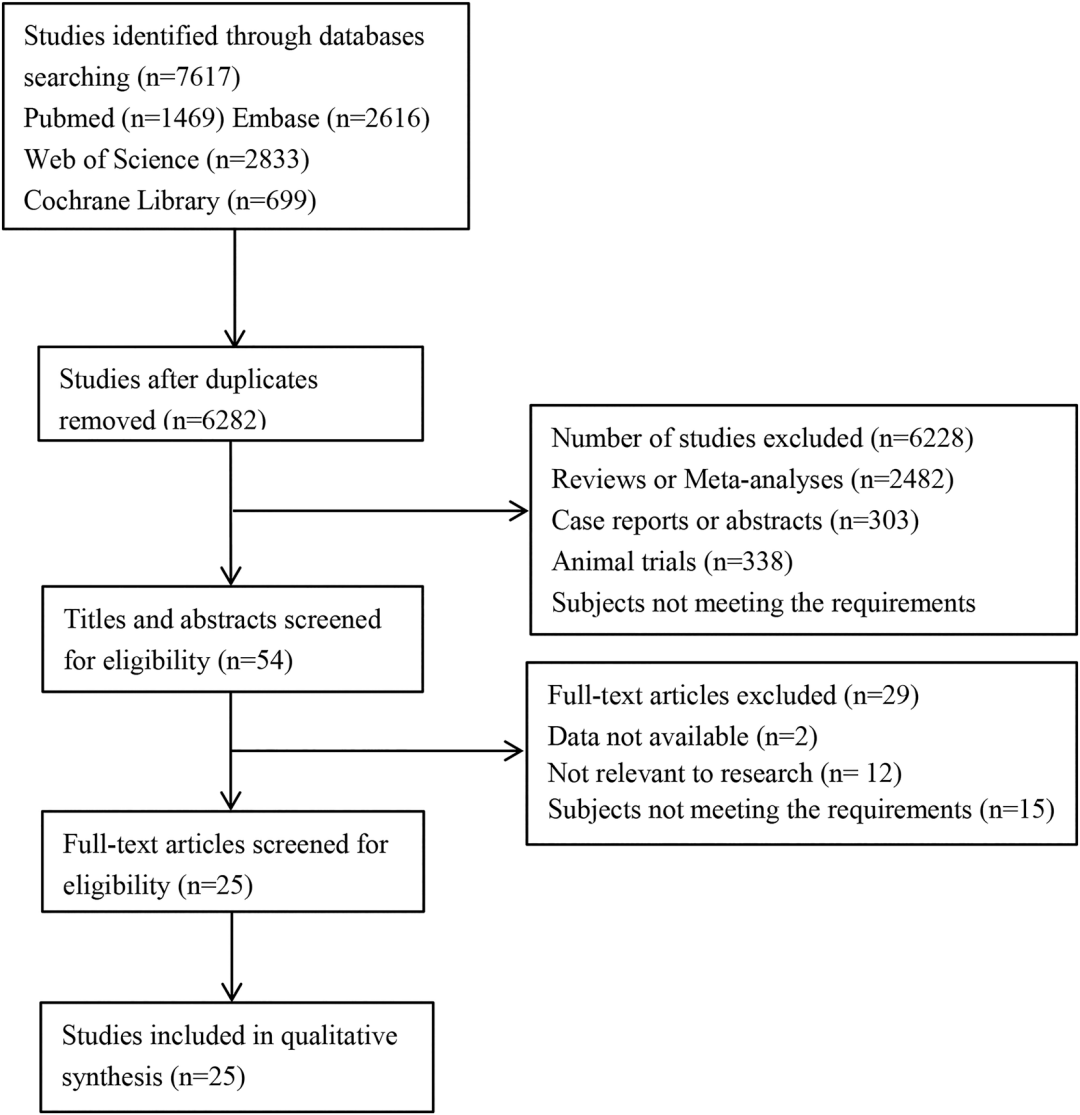
The literature search strategy of included studies

A total of 7617 articles were identified through databases searching, of which 6282 were duplicated articles. After title/abstract review, 54 records were selected. Finally, 25 articles [16, 18–41] including 54,000 patients met the established inclusion criteria and were enrolled in this study. The literature search strategy of included studies is depicted in Fig. 1. There were 10 studies from the USA, 7 studies from Europe, 6 studies from Asia, and 2 studies from Australia. The characteristics of these studies are presented in Table 1. And the risk of bias assessment of included studies is shown in Table 2.

**Table 1:** Characteristics of included studies

References	Author	Year	Location	Follow-up	Exposure assessment	Number of participants, sex, age	Total cases	T2D assessment	Exposure	Categories	RR (95% CI), HR [95% CI]	Adjustment factors
[18]	Alhazmi	2013	Australia	6 years	Validated dietary Questionnaire for Epidemiological Studies (DQES) version 2	8370, w, 45–50 years	311	Self-report, validated by linking to Medicare (MBS) and Pharmaceutical Benefits Scheme (PBS) databases for the years 2002–2005	PUFA	4.31 g/d (REF) 6.77 g/d 9.15 g/d 11.95 g/d 16.52 g/d	1.00 0.81 (0.53, 1.25) 1.10 (0.73, 1.67) 1.07 (0.71, 1.61) 1.27 (0.84, 1.90)	Area of residence, education, current smoking status, physical activity, self-rated health as good, menopausal status, BMI, alcohol consumption, total energy intake (kj/d), SFA and MUFA intakes for total carbohydrate, SFA, MUFA and fibre intakes for total protein, and fibre intake for total fat
									Total omega-3	0.61 g/d (REF) 0.86 g/d 1.08 g/d 1.37 g/d 1.97 g/d	1.00 0.98 (0.63, 1.52) 1.27 (0.84, 1.92) 1.44 (0.97, 2.16) 1.55 (1.03, 2.32)	
									EPA + DHA	0.09 g/d (REF) 0.17 g/d 0.25 g/d 0.38 g/d 0.73 g/d	1.00 1.07 (0.71. 1.60) 1.16 (0.77, 1.75) 1.12 (0.75, 1.68) 1.23 (0.84, 1.80)	
									EPA	0.02 g/d (REF) 0.04 g/d 0.07 g/d 0.12 g/d 0.24 g/d	1.00 1.06 (0.71. 1.59) 1.19 (0.79, 1.79) 1.07 (0.71, 1.62) 1.24 (0.85, 1.82)	
									DHA	0.06 g/d (REF) 0.11 g/d 0.17 g/d 0.26 g/d 0.49 g/d	1.00 1.04 (0.69, 1.55) 1.07 (0.71, 1.61) 1.10 (0.73, 1.64) 1.19 (0.81, 1.74)	
									ALA	0.42 g/d (REF) 0.61 g/d 0.78 g/d 0.98 g/d 1.40 g/d	1.00 1.20 (0.78, 1.82) 1.17 (0.76, 1.80) 1.32 (0.86, 2.01) 1.84 (1.25, 2.71)	
									Total omega-6	3.54 g/d (REF) 5.47 g/d 7.43 g/d 9.86 g/d 13.87 g/d	1.00 1.25 (0.83, 1.90) 1.18 (0.76, 1.83) 1.28 (0.82, 1.99) 1.60 (1.03, 2.48)	
[19]	Brostow	2011	China	5.7 years	Validated, semi-quantitative FFQ including 165 commonly consumed items	43,176, m/w, 45–74 years	2252	Self-reported, validation study of incident diabetes mellitus cases	Total omega-3	0.45 g/d (REF) 0.66 g/d 0.82 g/d 1.02 g/d 1.54 g/d	1.00 0.87 [0.75, 1.00] 0.88 [0.76, 1.02] 0.80 [0.68, 0.94] 0.78 [0.65, 0.94]	Age, sex, dialect, year of interview, educational level, BMI, physical activity, smoking status, alcohol use, hypertension, intakes of omega-6 or omega-3, MUFA, SFA, dietary fiber, protein, and total energy
									Total omega-6	3.50 g/d (REF) 5.40 g/d 7.10 g/d 9.30 g/d 14.60 g/d	1.00 0.94 [0.81, 1.08] 1.00 [0.87, 1.17] 0.91 [0.78, 1.07] 0.93 [0.87, 1.12]	
									EPA and DHA	0.11 g/d (REF) 0.22 g/d 0.30 g/d 0.38 g/d 0.60 g/d	1.00 1.01 [0.88, 1.17] 0.99 [0.85, 1.14] 0.94 [0.80, 1.10] 0.93 [0.77, 1.11]	
									ALA	0.27 g/d (REF) 0.40 g/d 0.51 g/d 0.65 g/d 1.06 g/d	1.00 0.91 [0.80, 1.04] 0.81 [0.70, 0.93] 0.78 [0.67, 0.90] 0.79 [0.67, 0.93]	
[20]	Djoussé	2011	USA	12.4 years	Validated baseline 128-FFQ	36,328, w, 54.6 years	2370	Self-report, validated using the ADA criteria (additional information via telephone interview and supplemental questionnaire)	ALA	0.79 g/d (REF) 0.96 g/d 1.11 g/d 1.29 g/d 1.59 g/d	1.00 0.94 [0.82, 1.09] 0.98 [0.85, 1.14] 1.00 [0.86, 1.17] 1.01 [0.85, 1.21]	Age, BMI, parental history of diabetes, smoking, exercise, alcohol intake, menopausal state, red-meat intake, quintiles of energy intake, linoleic acid, a-linolenic acid, dietary magnesium, trans fat, saturated fat, cereal fiber, and glycemic index
									EPA	0.01 g/d (REF) 0.02 g/d 0.03 g/d 0.08 g/d 0.12 g/d	1.00 1.08 [0.94, 1.24] 1.25 [1.11, 1.42] 1.30 [1.13, 1.49] 1.38 [1.21, 1.59]	
									DHA	0.04 g/d (REF) 0.09 g/d 0.12 g/d 0.17 g/d 0.17 g/d	1.00 1.21 [1.06, 1.38] 1.21 [1.06, 1.39] 1.46 [1.28, 1.68] 1.52 [1.33, 1.75]	
									Marine-n3	0.07 g/d (REF) 0.13 g/d 0.18 g/d 0.28 g/d 0.43 g/d	1.00 1.17 [1.03, 1.33] 1.20 [1.05, 1.38] 1.46 [1.28, 1.66] 1.44 [1.25, 1.65]	
[21]	Djoussé	2011	USA	9.6 years	Validated picture-sort FFQ in 1989–1990 and a FFQ (1995–1996 examination)	3088, m/w, 75.6 years for men, 74.7 years for women	204	(1) The new use of insulin or oral hypoglycemic agents, (2) A Fasting glucose concentration ≥ 7 mmol/l (126 mg/dl), or (3) a Nonfasting glucose concentration ≥ 11.1 mmol/l (200 mg/dl)	EPA + DHA	0.105 g/d (REF) 0.235 g/d 0.430 g/d 0.690 g/d	1.00 1.11 (0.74, 1.66) 0.78 (0.50, 1.22) 1.04 (0.67, 1.60)	Age, race (black or nonblack), sex, clinic site, BMI, alcohol consumption, physical activity, current smoking, LDL cholesterol, and linoleic acid
									ALA	0.095 g/d (REF) 0.125 g/d 0.160 g/d 0.200 g/d	1.00 0.82 (0.51, 1.33) 0.99 (0.56, 1.77) 0.50 (0.24, 1.05)	
[22]	Dow	2016	France	18 years	Validated 208-item FFQ	71,334, w, 52.9 years	2610	Self-report or reimbursements from health insurance records at least once between January 2004 and March 2012 Additional questionnaire → cases validated, if one of the following criteria was met: (1) fasting plasma glucose ≥ 7.0 mmol/l, (2) random glucose ≥ 11.1 mmol/l at diagnosis, (3) report of diabetic medication use. (4) Or last values of fasting glucose or hba1c concentrations ≥ 7.0 mmol/l o ≥ 7%, respectively	PUFA	< 12.0 g/d (REF) 12.0–15.3 g/d ≥ 15.3 g/d	1.00 1.03 [0.93, 1.14] 1.06 [0.96, 1.17]	Daily energy intake, alcohol consumption, level of education, family history of diabetes, physical activity, hypertension, Hypercholesterolaemia, smoking status, tertile groups of remaining fatty acid groups and BMI (age as time-scale in cox regression model)
									Total omega-3	< 1.3 g/d (REF) 1.3–1.6 g/d ≥ 1.6 g/d	1.00 1.10 [0.99, 1.22] 1.26 [1.13, 1.41]	
									EPA	< 0.09 g/d (REF) 0.09–0.20 g/d ≥ 0.20 g/d	1.00 0.88 [0.73, 1.06] 0.88 [0.67, 1.15]	
									DHA	< 0.19 g/d (REF) 0.19–0.38 g/d ≥ 0.38 g/d	1.00 1.15 [0.95, 1.38] 1.11 [0.85, 1.44]	
									ALA	< 0.90 g/d (REF) 0.90–1.14 g/d ≥ 1.14 g/d	1.00 1.00 [0.90, 1.12] 1.03 [0.92, 1.15]	
									Total omega-6	< 10.5 g/d (REF) 10.5–13.7 g/d ≥ 13.7 g/d	1.00 1.01 [0.91, 1.12] 1.00 [0.90, 1.10]	
									LA	< 10.3 g/d (REF) 10.3–13.5 g/d ≥ 13.5 g/d	1.00 0.98 [0.89, 1.08] 0.97 [0.87, 1.07]	
									AA	< 0.19 g/d (REF) 0.19–0.25 g/d ≥ 0.25 g/d	1.00 1.11 [0.99, 1.24] 1.49 [1.33, 1.66]	
[23]	Ericson	2015	Sweden	14 years	Interview-based, modified diet history Method (validated): - 7-d menu book - 168-item FFQ - a 45-min Interview	26,930, m/w, 45–74 years	2860	Via at least one of 7 registries or at new screenings or examinations during follow-up Information on date of diagnosis was used from 2 registries (the regional Diabetes 2000 Registry of Scania and the Swedish National Diabetes Registry) that required a physician diagnosis According to established diagnosis criteria: fasting plasma glucose Concentration ≥ 7.0 mmol/l or fasting whole blood concentration ≥ 6.1 mmol/l, measured at two different occasions	PUFA	4 E% (REF) 5 E% 6 E% 7 E% 8 E%	1.00 1.08 [0.96, 1.22] 1.04 [0.92, 1.17] 1.08 [0.96, 1.22] 1.07 [0.95, 1.20]	Age, sex, method version, season, total energy intake, leisure-time physical activity, smoking, alcohol intake, education and BMI
									Total omega-3	0.7 E% (REF) 0.8 E% 0.9 E% 1.1 E% 1.4 E%	1.00 0.90 [0.80, 1.02] 0.91 [0.81, 1.02] 0.93 [0.83, 1.05] 1.00 [0.89, 1.12]	
									Long-chain omega-3	0.07 E% (REF) 0.12 E% 0.19 E% 0.29 E% 0.52 E%	1.00 1.01 [0.90, 1.14] 0.99 [0.88, 1.12] 0.92 [0.81, 1.04] 1.07 [0.94, 1.20]	
									ALA	0.5 E% (REF) 0.6 E% 0.7 E% 0.8 E% 1.0 E%	1.00 0.85 [0.76, 0.95] 0.94 [0.84, 1.05] 0.85 [0.76, 0.95] 0.94 [0.83, 1.05]	
									Total omega-6	3.2 E% (REF) 4.0 E% 4.7 E% 5.5 E% 6.8 E%	1.00 1.13 [1.00, 1.28] 1.07 [0.95, 1.21] 1.11 [0.98, 1.25] 1.09 [0.97, 1.23]	
[24]	Guasch-Ferre	2017	Spain	4.3 years	Validated semiquantitative FFQ, completed in a face-to-face interview by trained dieticians	3349, m/w, 55–80 years	266	T2D incidence diagnosed according to ADA criteria	PUFA	4.14 En% (REF) 5.20 En% 6.23 En% 8.28 En%	1.00 1.25 [0.81, 1.91] 1.32 [0.85, 2.05] 1.56 [1.03, 2.35]	Age, sex, intervention group, BMI, smoking status, educational status, leisure-time physical activity, baseline hypertension or use of antihypertensive medication, total energy intake, alcohol intake, quartiles of fiber, protein intake, dietary cholesterol, specific types of fat, hypercholesterolemia or use of lipid-lowering drugs and fasting plasma glucose at baseline
									Marine omega-3	0.14 En% (REF) 0.23 En% 0.32 En% 0.57 En%	1.00 1.28 [0.87, 1.88] 1.06 [0.69, 1.61] 1.10 [0.71, 1.72]	
									Nonmarine omega-3	0.35 En% (REF) 0.44 En% 0.55 En% 0.80 En%	1.00 1.20 [0.78, 1.84] 1.20 [0.75, 1.93] 1.19 [0.72, 1.97]	
									LA	3.24 En% (REF) 4.21 En% 5.20 En% 7.11 En%	1.00 1.46 [0.95, 2.25] 1.47 [0.91, 2.37] 1.59 [0.96, 2.63]	
[25]-a	Kaushik	2009	USA	29 years	Validated semi-quantitative FFQ, 120 items	61,031, w, 30–55 years	4159	Self-report, validated according to the National Diabetes Data Group criteria	Long-chain omega-3	0.06 g/d (REF) 0.12 g/d 0.18 g/d 0.27 g/d 0.49 g/d	1.00 1.00 (0.91, 1.11) 1.12 (1.02, 1.24) 1.17 (1.05, 1.29) 1.23 (1.11, 1.37)	Age, smoking, alcohol consumption, physical activity, family history of diabetes, BMI, intakes of SFA, TFA, ALA, LA, caffeine, cereal fiber, glycemic index, calories, menopausal status and postmenopausal hormone use
[25]-b	Kaushik	2009	USA	15 years	Validated semi-quantitative FFQ, 120 items	61,669, w, 26–46 years	2728	Self-report, validated according to the National Diabetes Data Group criteria	Long-chain omega-3	0.06 g/d (REF) 0.10 g/d 0.15 g/d 0.22 g/d 0.36 g/d	1.00 1.04 (0.92, 1.17) 1.08 (0.95, 1.22) 1.15 (1.02, 1.30) 1.25 (1.10, 1.42)	Age, smoking, alcohol consumption, physical activity, family history of diabetes, BMI, intakes of SFA, TFA, ALA, LA, caffeine, cereal fiber, glycemic index, calories, hormone replacement therapy and contraceptive use
[25]-c	Kaushik	2009	USA	18 years	Validated semi-quantitative FFQ, 120 items	42,504, m, 39–78 years	2493	Self-report, validated according to the National Diabetes Data Group criteria	Long-chain omega-3	0.09 g/d (REF) 0.18 g/d 0.28 g/d 0.39 g/d 0.62 g/d	1.00 1.00 (0.88, 1.13) 0.99 (0.87, 1.12) 1.11 (0.98, 1.26) 1.12 (0.98, 1.28)	Age, smoking, alcohol consumption, physical activity, family history of diabetes, BMI, intakes of SFA, TFA, ALA, LA, caffeine, cereal fiber, glycemic index and calories
[26]	Kröger	2011	Germany	7 years	Self-administered validated FFQ	2714, m/w, 50 years	670	The prevalence of diabetes at baseline was evaluated by a physician who used information on self-reported medical diagnoses, Medication records, and dieting behavior. Uncertainties regarding a proper diagnosis at baseline were clarified with the participant or treating physician	PUFA	11.6 E% fat (REF) 14.8 E% fat 17.5 E% fat 20.2 E% fat 24.5 E% fat	1.00 1.01 (0.71, 1.43) 1.30 (0.91, 1.86) 1.21 (0.86, 1.72) 1.26 (0.89, 1.77)	Age, sex, BMI, waist circumference, cycling, sports activity, education, smoking status, alcohol intake, occupational activity, coffee intake (energy adjusted), fiber intake (energy adjusted), total fat intake, and total energy intake
									Long-chain omega-3	0.04 E% fat (REF) 0.16 E% fat 0.23 E% fat 0.32 E% fat 0.59 E% fat	1.00 1.01 (0.71, 1.46) 0.82 (0.58, 1.18) 0.97 (0.69, 1.36) 1.29 (0.95, 1.75)	
									ALA	1.4 E% fat (REF) 1.7 E% fat 1.9 E% fat 2.1 E% fat 2.6 E% fat	1.00 1.13 (0.80, 1.59) 1.27 (0.90, 1.80) 1.31 (0.93, 1.85) 1.13 (0.80, 1.59)	
									LA	9.0 E% fat (REF) 12.1 E% fat 14.8 E% fat 17.4 E% fat 21.8 E% fat	1.00 0.90 (0.63, 1.28) 1.14 (0.80, 1.63) 1.08 (0.76, 1.54) 1.11 (0.79, 1.56)	
[27]	Meyer	2001	USA	11 years	Validated 127-item FFQ	35,988, w, 55–69 years	1890	Self-report, with validation of 85 cohort participants in 1988	PUFA	8.9 g/d (REF) 9.2 g/d 10.4 g/d 12.2 g/d 16.6 g/d	1.00 0.94 (0.81, 1.08) 0.91 (0.78, 1.06) 0.85 (0.73, 0.99) 0.88 (0.76, 1.02)	Age, total energy, WHR, BMI, physical activity, cigarette smoking, alcohol consumption, education, marital status, residential area, hormone replacement therapy, dietary magnesium and cereal fiber
									Long-chain omega-3	0.03 g/d (REF) 0.09 g/d 0.13 g/d 0.20 g/d 0.39 g/d	1.00 0.98 (0.84, 1.14) 1.01 (0.87, 1.18) 0.99 (0.85, 1.15) 1.20 (1.03, 1.39)	
[28]	Salmeron	1997	USA	6 years	Validated semi-quantitative 131-item FFQ	42,759, m, 40–75 years	523	According the criteria of NIDDM proposed by the National Diabetes Data Group (1979) and the World Health Organization (1985)	PUFA	9.2 g/d (REF) 11.3 g/d 12.8 g/d 14.5 g/d 17.4 g/d	1.00 1.00 (0.76, 1.34) 1.00 (0.76, 1.34) 1.16 (0.89, 1.54) 1.01 (0.77, 1.35)	Age, BMI, alcohol intake, smoking status, physical activity and family history of diabetes
[29]	Salmeron	2001	USA	14 years	Validated semi-quantitative FFQ including 61-items (1980) and 116–136 items (1840 and on)	84,204, w, 30–55 years	2507	According the criteria of NIDDM Proposed by the National Diabetes Data Group (1979) and the World Health Organization (1985)	PUFA	2.9 E% (REF) 3.4 E% 4.1 E% 4.8 E% 6.2 E%	1.00 0.86 (0.76, 0.97) 0.77 (0.67, 0.88) 0.75 (0.65, 0.86) 0.75 (0.65, 0.88)	Age, time period, BMI, cigarette smoking, parental history of diabetes, alcohol consumption, physical activity, percentage of energy from protein, total energy intake and dietary cholesterol
[30]	Song	2004	USA	8.8 years	Validated, semi-quantitative FFQ	37,309, w, 53.5–54.6 years	1558	Self-report, validation in subgroups via blood samples and telephone interviews according to the ADA criteria	Omega-3	0.95 g/d (REF) 1.17 g/d 1.34 g/d 1.54 g/d 1.88 g/d	1.00 1.09 (0.92, 1.29) 1.06 (0.89, 1.25) 1.13 (0.96, 1.34) 1.10 (0.93, 1.30)	Age, BMI, total energy intake, smoking, exercise, alcohol use, family history of diabetes, fiber intake, glycemic load, magnesium and total fat
									Omega-6	7.35 g/d (REF) 9.12 g/d 10.5 g/d 12.0 g/d 14.5 g/d	1.00 1.06 (0.89, 1.26) 1.09 (0.91, 1.31) 1.04 (0.86, 1.25) 0.95 (0.78, 1.16)	
[31]	Van Dam	2002	USA	12 years	Validated semi-quantitative 131-item FFQ	42,504, m, 40–75 years	1321	Self-report, validation according to WHO criteria (1985)	LA	3.5 E% (REF) 4.4 E% 4.9 E% 5.6 E% 6.8 E%	1.00 0.99 (0.83, 1.18) 1.03 (0.86, 1.23) 1.06 (0.89, 1.26) 0.89 (0.74, 1.06)	Age, total energy intake, time period, physical activity, cigarette smoking, alcohol consumption, hypercholesterolemia, hypertension, family history of diabetes, cereal fiber, magnesium and BMI
									ALA	321 mg/d (REF) 396 mg/d 458 mg/d 533 mg/d 671 mg/d	1.00 1.03 (0.86, 1.23) 1.10 (0.92, 1.31) 1.00 (0.84, 1.20) 0.93 (0.78, 1.11)	
									Long-chain omega.3	80 mg/d (REF) 155 mg/d 250 mg/d 350 mg/d 570 mg/d	1.00 1.01 (0.85, 1.19) 0.95 (0.79, 1.13) 1.05 (0.88, 1.25) 1.01 (0.84, 1.21)	
[32]	Van Woudenbergh	2009	Netherlands	12.4 years	Validated semi-quantitative 170-item FFQ	4472, m/w, 67.2 years	463	Defined according to WHO (1999) and ADA criteria (1997)	Long-chain omega-3	23.8 mg/d (REF) 89.4 mg/d 236.8 mg/d	1.00 1.06 (0.84, 1.34) 1.05 (0.80, 1.38)	Age, sex, smoking, education level, intake of energy, alcohol, TFA, fiber, selenium, Vitamin D and cholesterol
[33]-a	Villegas	2011	China	8.9 years	Validated FFQ	64,193, w, 40–70 years	2262	Self-report and confirmation according to ADA criteria	Long-chain omega-3	0.02 g/d (REF) 0.04 g/d 0.07 g/d 0.11 g/d 0.20 g/d	1.00 0.90 (0.80, 1.00) 0.84 (0.75, 0.94) 0.87 (0.77, 0.98) 0.84 (0.74, 0.95)	Age, energy intake, WHR, BMI, smoking, alcohol consumption, physical activity, income level, education level, occupation, family history of diabetes, hypertension and dietary pattern
[33]-b	Villegas	2011	China	4.1 years	Validated FFQ	51,963, m, 40–74 years	833	Self-report and confirmation according to ADA criteria	Long-chain omega-3	0.02 g/d (REF) 0.04 g/d 0.07 g/d 0.11 g/d 0.20 g/d	1.00 0.95 (0.77, 1.17) 0.86 (0.69, 1.07) 0.96 (0.77, 1.19) 0.89 (0.70, 1.12)	Age, energy intake, WHR, BMI, smoking, alcohol consumption, physical activity, income level, education level, occupation, family history of diabetes, hypertension and dietary pattern
[34]	Virtanen	2014	Finland	19.3 years	4-day food record	2212, m, 42–60 years	422	Self-report, fasting plasma glucose ≥ 7.0 mmol/l or 2-h oral glucose tolerance test plasma glucose ≥ 11.1 mmol/l, record linkage	Long-chain omega-3	< 0.05 g/d (REF) 0.05–0.19 g/d 0.20–0.43 g/d > 0.43 g/d	1.00 0.80 [0.61, 1.06] 0.91 [0.70, 1.19] 0.85 [0.65, 1.12]	Age, examination year, BMI, family history of diabetes, smoking, education years, leisure-time physical activity, intake of alcohol, serum linoleic acid and energy
[35]	Wang	2015	USA	50,105 person-years (follow-up in years not available)	1989–1990: validated 99-item, picture sort FFQ, 1995–1996: validated 131-item self-administered FFQ	4207, m/w, ≥ 65 years	407	(1) The new use of insulin or oral hypoglycemic agents, (2) a Fasting glucose concentration ≥ 7 mmol/l (126 mg/dl), or 3) a Nonfasting glucose concentration ≥ 11.1 mmol/l (200 mg/dl)	ALA	< 1.02 g/d (REF) 1.02–1.41 g/d 1.42–1.83 g/d > 1.83 g/d	1.00 0.91 [0.69, 1.20] 1.09 [0.82, 1.45] 1.06 [0.79. 1.43]	Age, sex, race, education, enrolment site, smoking site, alcohol consumption, prevalence of physical activity, BMI, waist circumference, CVD, hypertension at baseline, total energy intake, dietary score that comprised Consumption of whole grains, fish, fruits and vegetables, nuts and seeds, red and processed meat, sugar-sweetened beverages, and fried potatoes
[36]	Zheng	2018	China	5.6 years	Validated FFQ	2671, m, w, 40–75 years	213	Defined according to ADA criteria	Long-chain omega-3	0.021 g/d (REF) 0.042 g/d 0.068 g/d 0.12 g/d	1.00 0.75 [0.50, 1.14] 0.87 [0.59, 1.29] 0.78 [0.52, 1.19]	Age, sex, BMI, WHR, physical activity, education, alcohol, smoking, household income, family history of diabetes, total energy intake, intake of dairy products, red and processed meat, fruits and vegetables, fasting blood glucose and erythrocyte total n-6 PUFA
									EPA	0.008 g/d (REF) 0.016 g/d 0.025 g/d 0.042 g/d	1.00 0.77 [0.51, 1.15] 0.85 [0.57, 1.27] 0.76 [0.50, 1.16]	
									DHA	0.011 g/d (REF) 0.024 g/d 0.039 g/d 0.067 g/d	1.00 0.77 [0.51, 1.17] 0.87 [0.59, 1.30] 0.74 [0.49, 1.13]	
									ALA	0.49 g/d (REF) 0.66 g/d 0.84 g/d 1.19 g/d	1.00 1.37 [0.90, 2.09] 1.11 [0.72, 1.71] 1.53 [1.01, 2.33]	
[37]-a	Zong	2019	USA	32 years	Validated FFQ	83,648, w, 30–55 years	9375	Self-report, validated according to the National Diabetes Data Group criteria	Omega-6	2.62 E% (REF) 3.47 E% 4.16 E% 4.95 E% 6.32 E%	1.00 0.95 [0.89, 1.02] 1.00 [0.93, 1.07] 0.94 [0.87, 1.01] 0.97 [0.90, 1.06]	Age, ethnicity, smoking status, alcohol intake, family history of diabetes, menopausal status and postmenopausal hormone use, physical activity, multivitamin use, baseline hypertension, baseline hypercholesterolemia, updated BMI, total energy intake, intake of fruits and vegetable, total fat, *trans* fats, monounsaturated fats, other PUFAs Age, ethnicity, smoking status, alcohol intake, family history of diabetes, menopausal status and postmenopausal hormone use, physical activity, multivitamin use, baseline hypertension, baseline hypercholesterolemia, updated BMI, total energy intake, intake of fruits and vegetable, total fat, *trans* fats, monounsaturated fats, other PUFAs
									Linoleic acid	2.54 E% (REF) 3.39 E% 4.07 E% 4.86 E% 6.23 E%	1.00 0.96 [0.89, 1.02] 0.99 [0.93, 1.07] 0.96 [0.89, 1.03] 0.98 [0.91, 1.06]	
[37]-b	Zong	2019	USA	32 years	Validated FFQ	88,610, w, 25–44 years	5460	Self-report, validated according to the National Diabetes Data Group criteria	Omega-6	3.41 E% (REF) 4.17 E% 4.76 E% 5.43 E% 6.60 E%	1.00 0.93 [0.85, 1.02] 0.91 [0.82, 1.00] 0.94 [0.85, 1.04] 0.91 [0.80, 1.02]	
									Linoleic acid	3.33 E% (REF) 4.08 E% 4.68 E% 5.35 E% 6.51 E%	1.00 0.95 [0.87, 1.04] 0.91 [0.82, 1.00] 0.94 [0.85, 1.04] 0.93 [0.82, 1.05]	
[37]-c	Zong	2019	USA	26 years	Validated FFQ	41,771, m, 40.75 years	3607	Self-report, validated according to the National Diabetes Data Group criteria	Omega-6	3.53 E% (REF) 4.43 E% 5.13 E% 5.91 E% 7.24 E%	1.00 0.86 [0.77, 0.96] 0.90 [0.80, 1.01] 0.82 [0.73, 0.92] 0.74 [0.65, 0.85]	Age, ethnicity, smoking status, alcohol intake, family history of diabetes, physical activity, multivitamin use, baseline hypertension, baseline hypercholesterolemia, updated BMI, total energy intake, intake of fruits and vegetable, total fat, tran*s* fats, monounsaturated fats, other PUFAs
									Linoleic acid	3.45 E% (REF) 4.35 E% 5.05 E% 5.83 E% 7.16 E%	1.00 0.87 [0.78, 0.97] 0.88 [0.79, 0.99] 0.83 [0.74, 0.94] 0.77 [0.67, 0.88]	
[38]	Hodge	2007	Australia	4 years	A Kodak Ektachem Analyzer and the World Health Organization criteria current at the time	3737, m/w, 36–72 years	346	A self-administered 121-item food-frequency questionnaire	Omega-6	2.456 g/d (REF) 4.912 g/d 7.368 g/d 9.824 g/d 12.280 g/d	1.00 1.27 (0.83, 1.85) 1.10 (0.71, 1.68) 1.49 (0.98, 2.27) 1.42 (0.93, 2.18)	Age, sex, country of birth, family history of diabetes, physical activity, alcohol intake, BMI, and waist-hip ratio
									Omega-3	0.27 g/d (REF) 0.54 g/d 0.81 g/d 1.08 g/d 1.35 g/d	1.00 1.10 (0.72, 1.69) 1.06 (0.69, 1.63) 0.88 (0.57, 1.36) 0.97 (0.63, 1.48)	
									PUFA	2.724 g/d (REF) 5.448 g/d 8.172 g/d 10.896 g/d 13.620 g/d	1.00 1.18 (0.78, 1.81) 0.91 (0.59, 1.41) 1.46 (0.97, 2.21) 1.29 (0.84, 1.97)	
[39]	Nanri	2011	Japan	5 years	A food-frequency questionnaire (FFQ)	22,921, m, 40–69 years	572	A self-administered questionnaire at the third survey	PUFA	11.6 g/d (REF) 12.3 g/d 12.9 g/d 14.2 g/d	1.00 0.84 (0.67, 1.07) 0.80 (0.62, 1.03) 0.73 (0.54, 1.00)	Age (y), study area (11 areas), BMI (in kg/m^2^; 21, 21–22.9, 23–24.9, 25–26.9, or 27), smoking status (never; past; current: ,20 or 20 cigarettes/d), alcohol consumption (nondrinker, occasional drinker, or drinker with a consumption of, 150, 150–299, 300–449, or 450 g ethanol/week for men; nondrinker, occasional drinker, or drinker with a consumption of, 150 or 150 g ethanol/week for women), family history of diabetes mellitus (yes or no), total physical activity (quartile, metabolic equivalent-h/d), history of hypertension (yes or no), total energy intake (kcal/d), coffee consumption (almost never or, 1, 1, or 2 cups/d), and intakes of calcium (mg/d), magnesium (mg/d), dietary fiber (g/d), vegetables (g/d), fruit (g/d), meat (g/d), and rice (g/d)
[40]	Mirmiran	2018	Iran	5.8 years	The food frequency questionnaire (FFQ)	2139, m/w, 20–70 years	143	Using anti-diabetic drugs, fasting plasma glucose (FPG) ≥ 126 mg/ or 2 h plasma glucose (2-h PG) ≥ 11.1 mmol/l	PUFA	3.33 E% 6.67 E% 10.00 E%	1.00 0.77 (0.48 -1.21) 0.45 (0.24 -0.93)	Age, diabetes risk score (SBP (mm Hg) < 120 (0 point), 120 < SBP < 140 (3 point) and SBP ≥ 140 (7 point); family history of diabetes (5 point); waist to height ratio (whtr): < 0.54 (0 point), 0.54–0.59 (6 Point) and ≥ 0.59 (11 point); TG/HDL-C: < 3.5 (0 point) and ≥ 3.5 (3 point); FSG (mmol/l): < 5 (0 point), 50–5.5 (12 Point) and 5.6–6.9 (33 point), energy intake, total fiber and magnesium
									Omega-3	3.33 E% 6.67 E% 10.00 E%	1.00 0.86 (0.56 -1.34) 0.55 (0.31 -0.88)	
									LA	3.33 E% 6.67 E% 10.00 E%	1.00 1.00 (0.59–1.70) 0.72 (0.36–1.42)	
									ALA	3.33 E% 6.67 E% 10.00 E%	1.00 0.69 (0.41–1.18) 0.71 (0.37–1.38)	
[16]	Zhang	2019	China	14 years	Updated versions of Chinese Food Composition Table (FCT)	7069, m, ≥ 20 years	492	Both self-reported and plasma Glucose/hba1c diagnosed	Omega-3	0 mg/day (REF) 0–23.1 mg/day 23.1–73.8 mg/day ≥ 73.8 mg/day	1.00 1.50 (1.11–2.01) 1.66 (1.20–2.28) 1.74 (1.22–2.47)	Age, marital status, BMI, income, education, physical activity, smoking, alcohol use, hypertension, north–south position (north or south), site (urban or rural), intake of total energy, fruit, vegetable, protein, saturated fat, monounsaturated fat, omega-6 fatty acids, and α-linolenic acid
[41]	Øyen	2021	Norway	7.5 years	A validated 255-item semiquantitative food-frequency questionnaire (FFQ)	60,831, w, 31 years	683	Moba questionnaire, MBRN, and use of Medications noted in the norpd during pregnancy	Long-chain omega-3	0 g/d (REF) < 0.40 g/d ≥ 0.40 g/d	1.00 0.95 (0.83–1.09) 1.08 (0.95–1.23)	Energy intake, age, prepregnancy BMI, gestational diabetes mellitus, and gestational hypertension including preeclampsia

*FFQ* food frequency questionnaire, *BMI* body mass index, *PUFAs* polyunsaturated fatty acids, *ALA* alpha linolenic acid, *EPA* eicosapentaenoic acid, *DHA* docosahexaenoic acid, *E%* percent of total energy intake, *E%* fat: energy percent of total fat intake, *ADA* American Diabetes Association, *WHR* waist-to-hip ratio

**Table 2:** Risk of bias in non-randomized studies of interventions quality assessment

Study	Bias due to confounding	Bias due to selection of participants	Bias due to exposure assessment	Bias due to misclassification during follow-up	Bias due to missing data	Bias due to measurement of the outcome	Bias due to selective reporting of the results	Overall judgement
Alhazmi 2013	Moderate	Moderate	Moderate	Moderate	Low	Moderate	Low	Moderate
Brostow 2011	Moderate	Low	Moderate	Moderate	Low	Moderate	Low	Moderate
Djoussé 2011a (WHS)	Moderate	Low	Moderate	Moderate	Low	Moderate	Low	Moderate
Djoussé 2011b (CHS)	Serious	Moderate	Moderate	Low	Low	Low	Low	Serious
Dow 2016	Moderate	Low	Moderate	Moderate	Moderate	Moderate	Low	Moderate
Ericson 2015	Moderate	Moderate	Moderate	Moderate	Low	Low	Low	Moderate
Guasch-Ferre 2017	Moderate	Moderate	Moderate	Low	Low	Low	Low	Moderate
Kaushik 2009	Moderate	Low	Moderate	Low	No information	Moderate	Low	Moderate
Kröger 2011	Moderate	Low	Moderate	Moderate	Low	Low	Low	Moderate
Meyer 2001	Moderate	Moderate	Moderate	Moderate	Low	Moderate	Low	Moderate
Salmeron 1997	Moderate	Moderate	Moderate	Moderate	Low	Moderate	Low	Moderate
Salmeron 2001	Moderate	Moderate	Moderate	Moderate	No information	Low	Low	Moderate
Song 2004	Moderate	Moderate	Moderate	Moderate	Low	Moderate	Low	Moderate
Van Dam 2002	Moderate	Moderate	Moderate	Low	Low	Moderate	Low	Moderate
Van Woudenbergh 2009	Moderate	Moderate	Moderate	Moderate	Moderate	Low	Low	Moderate
Villegas 2011	Moderate	Low	Moderate	Low	Low	Low	Low	Moderate
Virtanen 2014	Moderate	Low	Serious	Moderate	Low	Low	Low	Serious
Wang 2015	Moderate	Low	Moderate	Low	Low	Low	Low	Moderate
Zheng 2018	Moderate	Low	Moderate	Moderate	Moderate	Low	Low	Moderate
Zong 2019	Moderate	Moderate	Moderate	Low	Low	Moderate	Low	Moderate
Hodge 2007	Moderate	Moderate	Moderate	Moderate	Low	Moderate	Low	Moderate
Nanri 2011	Moderate	Moderate	Moderate	Moderate	Low	Moderate	Low	Moderate
Mirmiran 2018	Moderate	Low	Moderate	Moderate	Moderate	Moderate	Low	Moderate
Zhang 2019	Moderate	Low	Moderate	Moderate	Low	Low	Low	Moderate
Øyen 2021	Moderate	Moderate	Moderate	Low	Low	Moderate	Low	Moderate

### PUFA intake and incidence of T2D

A total of 25 articles were included to assess the association between PUFA intake and the incidence of T2D. The heterogeneity test showed that I^2^ = 68.2%, so the random-effect model was used for analysis. The result demonstrated that total PUFA intake could not be considered to be associated with the development of T2D (RR: 1.012, 95% CI 0.992 to 1.032, *P* = 0.246) (Table 3).

**Table 3:** Overall results and sensitivity analysis

Indicators	Summary RR (95% CI)	*p*	I^2^ (%)
Total PUFA	1.012 (0.992, 1.032)	0.246	68.2
Sensitivity analysis	1.012 (0.992, 1.032)		
Publication bias	Z = 2.46	0.014	
Sex			
Women	1.049 (1.019, 1.079)	0.001	77.1
Men	0.955 (0.913, 0.999)	0.044	62.2
Mixed	0.984 (0.955, 1.013)	0.269	41.2
Geographic location			
United States	1.011 (0.982, 1.041)	0.467	78.2
Europe	1.040 (1.009, 1.072)	0.012	54.8
Australia	1.188 (1.113, 1.269)	< 0.001	0.0
Asia	0.897 (0.860, 0.936)	< 0.001	45.4
Duration of follow-up, y			
< 10	0.999 (0.968, 1.031)	0.942	38.4
≥ 10	1.016 (0.991, 1.042)	0.200	79.2
Exposure			
PUFA	0.979 (0.905, 1.058)	0.588	63.7
Omega-3	1.028 (0.987,1.070)	0.183	69.0
Omega-6	0.985 (0.942, 1.030)	0.511	60.7
ALA	1.003 (0.966, 1.041)	0.887	59.2
EPA	1.078 (0.965, 1.203)	0.183	64.9
DHA	1.164 (1.048, 1.294)	0.005	61.4
EPA + DHA	0.992 (0.926, 1.064)	0.830	0.0
LA	0.956 (0.930, 0.983)	0.001	40.4
AA	1.286 (0.964, 1.716)	0.087	92.5

*RR* relative risk, *PUFA* polyunsaturated fatty acid, *ALA* alpha linolenic acid, *EPA* eicosapentaenoic acid, *DHA* docosahexaenoic acid, *LA* linoleic acid, *AA* arachidonic acid

Based on sex subgroup, consumption of total PUFA would increase the incidence of T2D among women (I^2^ = 77.1%, RR: 1.049, 95% CI 1.019 to 1.079, *P* = 0.001), while decreasing the incidence of T2D among men (I^2^ = 62.2%, RR: 0.955, 95% CI 0.913 to 0.999, *P* = 0.044) (Table 3).

When concerning geographic location, total PUFA intake was associated with increased incidence of T2D in Europe (I^2^ = 54.8%, RR: 1.040, 95% CI 1.009 to 1.072, *P* = 0.012), and Australia (I^2^ = 0.0%, RR: 1.188, 95% CI 1.113 to 1.269, *P* < 0.001). However, total PUFA intake decreased the incidence of T2D in Asia (I^2^ = 45.4%, RR: 0.897, 95% CI 0.860 to 0.936, *P* < 0.001) (Table 3).

Subgroup analysis based on the duration of follow-up indicated that there was no association between total PUFA intake and T2D when the duration of follow-up was < 10 years (I^2^ = 38.4%, RR: 0.999, 95% CI 0.968 to 1.031, *P* = 0.942), and ≥ 10 years (I^2^ = 79.2%, RR: 1.016, 95% CI 0.991 to 1.042, *P* = 0.200) (Table 3).

Subgroup analysis based on PUFA types indicated that no association with T2D incidence for omega-3 PUFA (I^2^ = 69.0%, RR: 1.028, 95% CI 0.987 to 1.070, *P* = 0.183), omega-6 PUFA (I^2^ = 60.7%, RR: 0.985, 95% CI 0.942 to 1.030, *P* = 0.511), ALA (I^2^ = 59.2%, RR: 1.003, 95% CI 0.966 to 1.041, *P* = 0.887), EPA (I^2^ = 64.9%, RR: 1.078, 95% CI 0.965 to 1.203, *P* = 0.183), and AA (I^2^ = 92.5%, RR: 1.286, 95% CI 0.964 to 1.716, *P* = 0.087). Consumption of DHA was associated with T2D incidence (I^2^ = 61.4%, RR: 1.164, 95% CI 1.048 to 1.294, *P* = 0.005). However, lower T2D incidence was observed with LA intake (I^2^ = 40.4%, RR: 0.956, 95% CI 0.930 to 0.983, *P* = 0.001) (Table 3).

### Dose–response relationship between PUFA and incidence of T2D

**Fig. 2:**
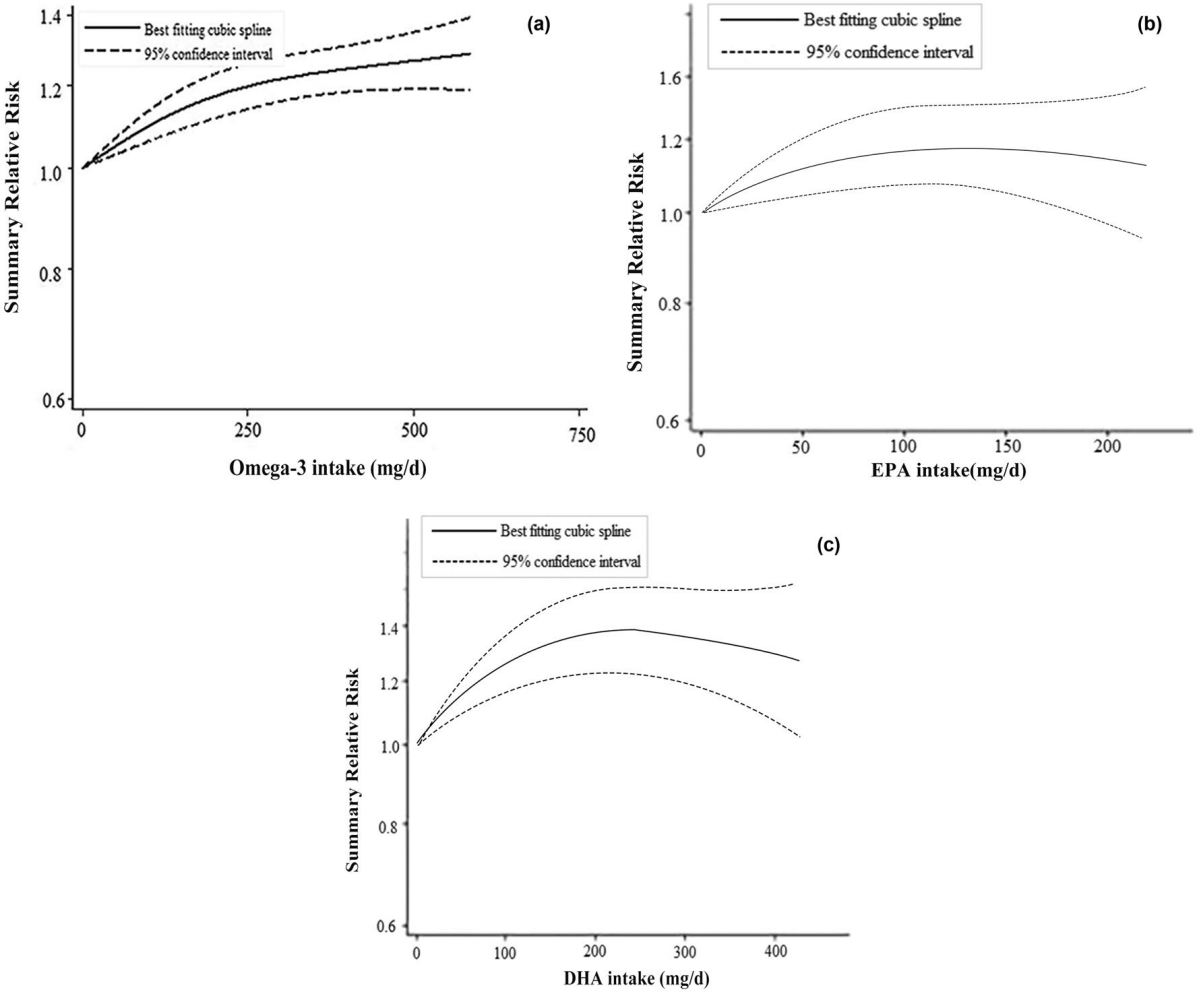
Diagram of nonlinear dose response association between PUFA and incidence of T2D; **a** omega-3 PUFA; **b** EPA; **c** DHA. **a** *P*_nonlinearity_ < 0.001; **b** *P*_nonlinearity_ = 0.023; **c** *P*_nonlinearity_ = 0.040

We observed no linear associations between PUFA and the incidence of T2D. Thus, we summarized the non-linear dose–response of different types of PUFA to the incidence of T2D. The dose–response relationship showed an increasing nonlinear trend as the accumulated omega-3 PUFA intake increased (*P*_nonlinearity_ < 0.001) (Fig. 2a) while there were no significant nonlinear associations between total PUFA, omega-6 PUFA, ALA, LA intakes and T2D incidence. When EPA intake was between 110 and 150 mg/d, an increasing nonlinear trend of T2D incidence was observed (*P*_nonlinearity_ = 0.023), after which the curve decreased slightly, remaining close to no association (Fig. 2b). The T2D risk was highest when DHA intake was 200–300 mg/d, and the dose–response association was statistically significant (*P*_nonlinearity_ = 0.040) (Fig. 2c).

### Sensitivity analysis and publication bias

**Fig. 3:**
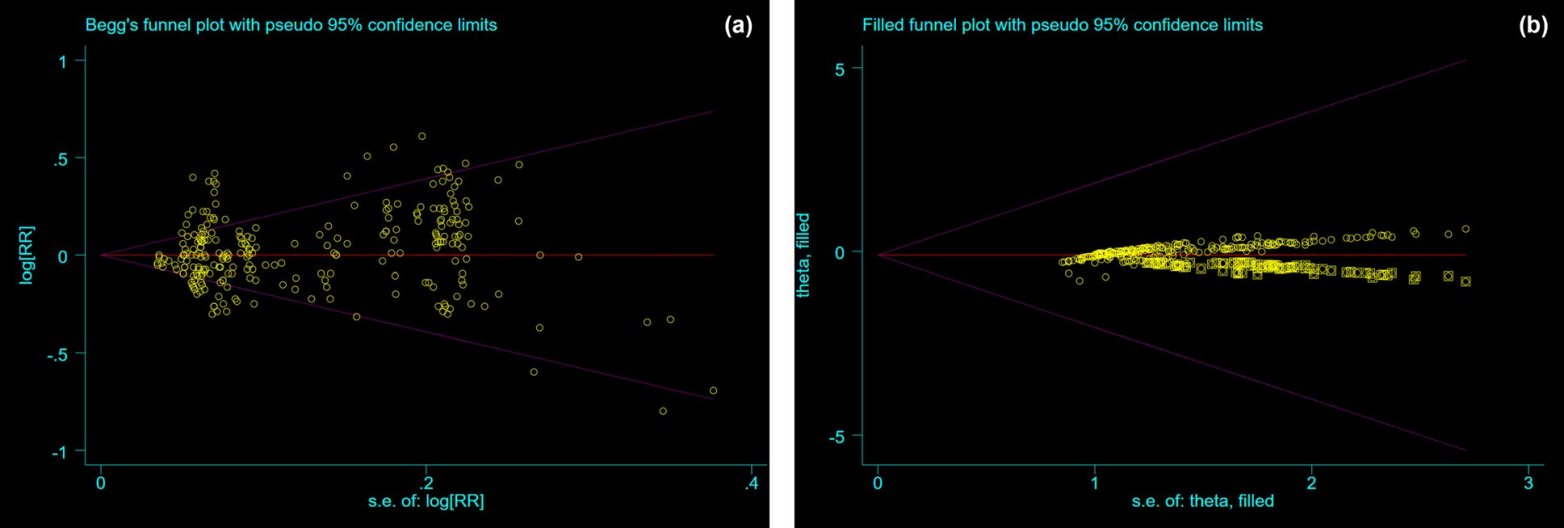
Begg’s funnel plot of publication bias; **a** unadjusted; **b** adjusted. **a** RR = 1.012, 95% CI 0.992 to 1.032; **b** RR = 0.903, 95% CI 0.785 to 1.038

Our sensitivity analysis suggested the stability of the result, indicating our findings are robust (Table 3). Begg’s test result showed that there was a publication bias in this study, so the “cut-and-fill method” was adopted to adjust the bias and effect size. The combined prevalence of the random effect model before the “cut-and-fill method” was 1.012 (95% CI 0.992 to 1.032). The estimated number of missing studies was 7. Then, all the studies were re-meta-analyzed after the studies with missing estimates were included. After the “cut-and-fill method”, the combined prevalence of the random effect model was 0.903 (95% CI 0.785 to 1.038) (Fig. 3).

## Discussion

The prevalence of T2D is rising sharply in nearly all nations in the world [42], which highlights the need for widespread preventive treatment. Of all the major guidelines, diet is the cornerstone of prevention and treatment [43]. Nevertheless, the association between PUFA and T2D incidence is inconclusive [5]. Thus, in this study, we estimated the associations and the dose–response relationship between the dose of PUFA intake and T2D development. We found that omega-3 PUFA and DHA intakes had nonlinear dose–response relationships with T2D incidence. Moreover, subgroup analysis suggested total PUFA intake was associated with increased incidence of T2D in Europe and Australia whereas it was associated with a decreased incidence in Asia. Regarding the type of PUFA, DHA intake was associated with an elevated T2D incidence, while LA was associated with a decreased incidence of T2D. Additionally, consumption of total PUFA would increase the incidence of T2D in women while decreasing the incidence of T2D in men. However, there was no linear association between PUFA intake and the incidence of T2D.

Omega fatty acids are PUFA with an acid end containing the functional carboxylic acid group and a methyl end, also known as the omega end. In omega-3 and omega-6 fatty acids, the first site of desaturation is located after the third and the sixth carbon from the omega end, respectively [44]. Omega-3 fatty acids are found in salmon, mackerel, and other cold-water fish, as well as flaxseed, walnuts, and canola oil [45]. DHA and EPA are long-chain omega-3 fatty acids that are present in fish oils, seafood, algae, and fortified foods, while ALA is derived from plant sources [37]. Our nonlinear dose–response meta-analysis indicated a significant association of increased T2D incidence with increasing omega-3 PUFA intake. A study [16] evaluating the current level of omega-3 PUFA intake and risk of T2D in China has found that intake of marine omega-3 PUFA was dose-dependently associated with higher T2D risk for both men and women. Dow et al. [22] found high omega-3 PUFA consumption was associated with T2D even after adjustment for confounders. The association between omega-3 PUFA and T2D risk may be due to the effects of mega-3 PUFA on blood glucose and insulin sensitivity. A high intake of omega-3 PUFA has been found to increase blood glucose and decrease insulin sensitivity [46]. In our dose–response analysis, further examination of the source of omega-3 PUFA revealed that a higher DHA intake was significantly associated with T2D incidence and the risk was highest when DHA intake reached 200–300 mg/d. Kaushik et al. examined the association between dietary omega-3 PUFA and incidence of T2D in 3 prospective cohorts of women and men [25], finding an increased risk of T2D with the intake of long-chain omega-3 PUFA (EPA and DHA) especially with higher intakes (200 mg/d). The result of our dose–response analysis indicates that the potentially detrimental effect of DHA or omega-3 PUFA intake threshold should be focused on and further studied.

LA is the predominant omega-6 PUFA [47] and accounts for 80–90% of total dietary PUFA, which was associated with a reduction in the incidence of T2D in this study. The protective effect of LA on insulin homeostasis has been well characterized [48]. In a consortium of 20 studies across ten countries, biomarker levels of LA were inversely associated with incident T2D; dietary PUFA (mostly LA) improved blood sugar, IR, and insulin secretion compared with carbohydrates, saturated fats, and even monounsaturated fats at some endpoints [49]. In the European Prospective Investigation into Cancer and Nutrition study, Forouhi et al. provided evidence of strong and significant inverse associations between T2D and LA; the risk decreased by 20% for every 1-standard deviation increased LA [50]. Based on U.S. data, Zong et al. provided additional evidence that LA intake was inversely associated with the risk of T2D [37]. Experimental evidence supports the biological plausibility of beneficial effects of omega-6 LA, on several mechanisms associated with insulin sensitivity and the development of T2D [51]. The incorporation of unsaturated fats improves cell membrane fluidity and function, such as glucose transporter (GLUT) translocation, insulin receptor binding and affinity, cell signal transduction, and ion permeability, which together improve insulin sensitivity [52].

We observed geographic differences regarding the association of T2D incidence and PUFA intake. An inverse association between long-chain omega-3 PUFA and T2D incidence was observed in the Asian population [7]. The protective association of omega-3 PUFA consumption with T2D was reported by the Japan Public Health Center-based Prospective Study in men and the Shanghai Women’s Health Study [33, 39]. Chen et al. and Wallin et al. concluded omega-3 PUFA intakes were related to lower T2D risk only in Asian but not North American or European populations [4, 53]. In Caucasians, cohort studies suggested an elevating T2D risk with the increase of fish and omega-3 PUFA intakes [20, 25]. The discrepancy in findings may be partly because an Asian population has different metabolic and lifestyle characteristics with T2D compared with the United States or European populations [54]. Western dietary patterns are characterized by high intake of sugar, red meat, and fried foods; The dietary pattern of Asians, especially The Chinese and Japanese, is also known as the prudent dietary pattern, which includes a high intake of fruits, vegetables, fish and tofu [36, 55]. These findings have important public health implications. The influence of genetic and gene–diet interactions on T2D in different populations needs to be further explored to understand the relationship between PUFA intake and T2D incidence.

We found that the consumption of total PUFA was associated with the risk of an increasing T2D in women while the consumption of total PUFA was associated with the risk of a decreasing incidence of T2D in men. However, there was no linear association between PUFA intake and the incidence of T2D. In a cohort of Australian women from the Australian Longitudinal Study of Women’s Health, total omega-3 PUFA, ALA, and total omega-6 PUFA intakes were positively associated with the incidence of T2D among women [18]. A cross-sectional study in China demonstrated that higher omega n-6 PUFA status may be protective against the risk of T2D in men [56]. In a prospective cohort comprising only 2189 middle-aged and older Finnish men, Yary et al. found that omega-6 PUFA was inversely associated with T2D in men but not in women [57]. Males and females differ in their levels of diabetes risk, which may attribute to the clear sex-specific disparities in dietary intake habits, which lead to varying PUFA profiles [58]. Sex differences may also be attributed to differences in the distribution and function of different adipose tissue depots in men and women [59].

This study is a detailed assessment of PUFA intake and T2D, including different classifications of PUFA, sex, duration of follow-up. The large sample size of the included studies makes this study more powerful to examine the associations between PUFA intake and T2D than any individual study. Besides, we further examined the linear and nonlinear association of PUFA intake and T2D incidence. However, potential limitations to this study should also be considered. Heterogeneity and potential publication bias may influence the result of this meta-analysis. The extent to which PUFA from different sources affects T2D development remains unknown. In our study, it is not clear whether the source of PUFA intake is food or supplements, and the relationship between source of PUFA and T2D needs to be elucidated in the future.

## Conclusions

In this study, omega-3 PUFA and DHA intakes had nonlinear dose–response associations with T2D incidence. PUFA was likely to have different effects on T2D incidence. In addition, regional and sex differences in the relationship between T2D and PUFA were also observed.

## Supplementary Information

**Additional file 1.** Detailed search strategy from PubMed.

## Abbreviations

T2D: Type 2 diabetes
PUFA: Polyunsaturated fatty acid
RR: Relative risk
HR: Hazard ratio
CI: Confidence interval
DHA: Docosahexaenoic acid
LA: Linoleic acid
IR: Insulin resistance
UFA: Unsaturated fatty acid
RCTs: Randomized controlled trials
ROBINS-I: Risk of bias in non-randomized studies of interventions
ALA: Alpha-linolenic acid
EPA: Eicosapentaenoic acid
AA: Arachidonic acid
REML: Restricted maximum likelihood

## Declarations

### Availability of data and materials

The datasets used and/or analyzed during the current study are available from the corresponding author on reasonable request.
