# Supplementary material for: Polyunsaturated fatty acid intake and incidence of type 2 diabetes in adults: a dose response meta-analysis of cohort studies
Source: Diabetol Metab Syndr. 2022 Mar 3;14:34. doi: 10.1186/s13098-022-00804-1 (PMC8892771; doi:10.1186/s13098-022-00804-1)
Supplement: Supplementary file 1 — Additional file 1. Detailed search strategy from PubMed. [file 13098_2022_804_MOESM1_ESM.docx]

The detailed search strategy from PubMed is as follows: “Fatty Acids, Unsaturated” OR “Acids, Unsaturated Fatty” OR “Unsaturated Fatty Acids” OR “Unsaturated Fatty Acid” OR “Acid, Unsaturated Fatty” OR “Fatty Acid, Unsaturated” OR “Polyunsaturated Fatty Acids” OR “Acids, Polyunsaturated Fatty” OR “Fatty Acids, Polyunsaturated” OR “Polyunsaturated Fatty Acid” OR “Acid, Polyunsaturated Fatty” OR “Fatty Acid, Polyunsaturated” OR “Fatty Acids” OR “Fatty Acid” OR “Fatty Acids, Esterified” OR “Esterified Fatty Acids” OR “Esterified Fatty Acid” OR “Acid, Esterified Fatty” OR “Fatty Acid, Esterified” OR “Fatty Acids, Saturated” OR “Saturated Fatty Acids” OR “Saturated Fatty Acid” OR “Acid, Saturated Fatty” OR “Fatty Acid, Saturated” OR “Aliphatic Acids” OR “Aliphatic Acid” OR “Acid, Aliphatic” AND “Diabetes Mellitus, Type 2” OR “Diabetes Mellitus, Noninsulin-Dependent” OR “Diabetes Mellitus, Ketosis-Resistant” OR “Diabetes Mellitus, Ketosis Resistant” OR “Ketosis-Resistant Diabetes Mellitus” OR “Diabetes Mellitus, Non Insulin Dependent” OR “Diabetes Mellitus, Non-Insulin-Dependent” OR “Non-Insulin-Dependent Diabetes Mellitus” OR “Diabetes Mellitus, Stable” OR “Stable Diabetes Mellitus” OR “Diabetes Mellitus, Type II” OR “NIDDM” OR “Diabetes Mellitus, Noninsulin Dependent” OR “Diabetes Mellitus, Maturity-Onset” OR “Diabetes Mellitus, Maturity Onset” OR “Maturity-Onset Diabetes Mellitus OR “Maturity Onset Diabetes Mellitus OR “MODY” OR “Diabetes Mellitus, Slow-Onset” OR “Diabetes Mellitus, Slow Onset” OR “Slow-Onset Diabetes Mellitus” OR “Type 2 Diabetes Mellitus” OR “Noninsulin-Dependent Diabetes Mellitus” OR “Noninsulin Dependent Diabetes Mellitus” OR “Maturity-Onset Diabetes” OR “Diabetes, Maturity-Onset” OR “Maturity Onset Diabetes” OR “Type 2 Diabetes” OR “Diabetes, Type 2” OR “Diabetes Mellitus, Adult-Onset” OR “Adult-Onset Diabetes Mellitus” OR “Diabetes Mellitus, Adult Onset” OR “Diabetes Mellitus”.
